# A Brief Review of In Situ and Operando Electrochemical Analysis of Bacteria by Scanning Probes

**DOI:** 10.3390/bios13070695

**Published:** 2023-06-30

**Authors:** Tzu-En Lin, Sorour Darvishi

**Affiliations:** 1 Institute of Biomedical Engineering, Department of Electrical and Computer Engineering, National Yang Ming Chiao Tung University, Hsinchu 30010, Taiwan; 2 Department of Electrical Engineering and Computer Sciences, University of California, Berkeley, CA 94720, USA; sorour.darvishi@berkeley.edu; 3 Berkeley Sensor and Actuator Center, University of California, Berkeley, CA 94720, USA

**Keywords:** scanning electrochemical microscopy, quorum sensing, metabolic activity, oxygen respiration, electrochemical active metabolites, oxygen consumption, pH value, biofilms, bacteria

## Abstract

Bacteria are similar to social organisms that engage in critical interactions with one another, forming spatially structured communities. Despite extensive research on the composition, structure, and communication of bacteria, the mechanisms behind their interactions and biofilm formation are not yet fully understood. To address this issue, scanning probe techniques such as atomic force microscopy (AFM), scanning electrochemical microscopy (SECM), scanning electrochemical cell microscopy (SECCM), and scanning ion-conductance microscopy (SICM) have been utilized to analyze bacteria. This review article focuses on summarizing the use of electrochemical scanning probes for investigating bacteria, including analysis of electroactive metabolites, enzymes, oxygen consumption, ion concentrations, pH values, biofilms, and quorum sensing molecules to provide a better understanding of bacterial interactions and communication. SECM has been combined with other techniques, such as AFM, inverted optical microscopy, SICM, and fluorescence microscopy. This allows a comprehensive study of the surfaces of bacteria while also providing more information on their metabolic activity. In general, the use of scanning probes for the detection of bacteria has shown great promise and has the potential to provide a powerful tool for the study of bacterial physiology and the detection of bacterial infections.

## 1. Introduction

The world faces a perilous global epidemic of antibiotic resistance that could undermine the very foundation of the healthcare system. The World Health Organization (WHO) has emphasized the crisis if antibiotics lose their effectiveness. Therefore, gaining a fundamental understanding of bacterial cell behavior is vital for medicine, the pharmaceutical industry, and alternative energy production. Bacterial colonies are highly structured and social communities that exist in complex and interdependent biofilms, which are tightly attached to living or non-living surfaces and embedded in a self-generated extracellular polymeric substance (EPS). Biofilms can adhere to living and non-living surfaces and display up to 1000 times greater resistance to antimicrobial agents than planktonic cell cultures [1]. 

The biofilm growth cycle starts with attachment to a surface and ends with the formation of mature, high-density bacterial communities. The colony releases free-swimming bacteria to the surrounding surface for attachment and growth [2]. They cooperate with each other and exchange beneficial compounds, ranging from cell-to-cell signals to iron-scavenging siderophores and digestive enzymes. However, it is important to note that not all biofilms are harmful. In fact, biofilms are widely used in microbial fuel cell (MFC) technology to convert the chemical energy of carbohydrates into electricity. Although biofilm research has gained increasing attention, our understanding of the spatial parameters that regulate their distribution and interactions between aggregates remains poor.

In recent decades, interest in utilizing scanning probe microscopies (SPMs) to study bacteria has grown. These techniques allow the creation of spatially resolved images of surfaces using a physical probe to scan the sample [3,4,5]. When image-related information is extracted, SPMs can reveal important physical structures or map specific biomolecules, providing deeper insight into prokaryotes and eukaryotes. Various SPM setups are available for investigating electrochemical systems, including many types such as scanning electrochemical microscopy (SECM), scanning ion-conductance microscopy (SICM), scanning electrochemical cell microscopy (SECCM), and atomic force microscopy (AFM) [6,7,8,9,10,11,12,13,14]. The scanning mechanism of SPMs is based on a probe that is scanned across a sample surface, and the probe–sample interaction is monitored by sensing changes in the local environment. Different SPMs utilize different types of electrodes or tips as probes and detect different types of probe–sample interactions as signals. This article summarizes the use of SPMs to study electrochemical reactions related to bacteria, with a particular focus on the advances in SECM and related technology. Continuing improvements in SECM and relevant techniques have led to many numerous innovative applications in the in situ and operando characterization of bio-surfaces, enzymes, catalysts screening, corrosion sites, biofilms, chemical kinetics, topographic changes, and instantaneous product analysis [13,15,16]. The adoption of biologically friendly scanning environments and versatile scanning probes (e.g., soft probes) has increased the popularity of SECM in the biological field (e.g., 1 atm, controllable temperature, and measurement in buffered solutions) [12,17,18,19,20,21,22,23]. Recent developments in soft SECM probes also expand the applications of traditional SECM, allowing the scanning of soft animal tissues, contact lenses, and fragile self-assembling layers [17,20,21,24,25,26,27,28,29,30].

## 2. The Basic Instrumentation of SECM

A typical SECM uses a working electrode as a probe that scans in the vicinity of a substrate interface, offering a map or line scan of the localized reactivity based on the sample–probe interaction under different operating modes. The SECM probe is usually a micro-electrode or nano-electrode connected to a motor positioning system that can precisely control the probe to move in the x-, y-, and z-directions [31]. A potentiostat is used to control the working potential at the probe (often also denoted as the tip “T”) acting as the working electrode (WE) versus a reference electrode. In addition, bipontentiostats can also be used to bias the substrate immersed in the electrolytes and redox mediators. In recent years, nano-electrodes have attracted much attention since they enhance the imaging resolution but require a smaller probe sample distance and hence more advanced instrumentation. In general, the resolution of the SECM is mainly determined by the size of the electrode, the working distance, and the sensitivity of the chosen operation mode (vide supra).

## 3. The Recent Development of Using Scanning Probes for Bacteria Analysis

Table 1 presents a recent compilation of studies that employ SECM-based tools to analyze bacteria or biofilms. The information in Table 1 encompasses the research goals, representative parameters, redox mediators, or solutions used in the experiments; more importantly, the information that could be ‘read’ by relevant scanning probe microscopy techniques. As shown in Table 1, SECM enables the quantitative detection and mapping of redox-active or charged molecules produced by bacteria in three dimensions with a micrometer or nanometer spatial resolution. Therefore, relevant SECM-based studies associated with bacteria, electroactive metabolites, enzymes, oxygen consumption, ion concentration, pH value, biofilms, and quorum sensing (QS) molecules could be investigated and will be introduced in the following sections. Scanning probe microscopy such as SECM offers advantages over optical measurements, as it is exclusively dependent on electrochemical signals, thus avoiding optical interference (e.g., sample color background). Furthermore, the chemical reaction between the probe and the substrate can be used to modify the surface of the sample and create nanostructures or microstructures by depositing or etching [32,33]. 

In addition to basic SECM, SECM combined with other techniques, such as AFM, inverted optical microscope, automatic motion control system, constant temperature chamber, SICM, or even fluorescence microscopy, has been developed. These advances will be discussed in the final part of this article. Finally, the contemporary and future perspectives of the SECM analysis of bacteria will be described.

**Figure 1:**
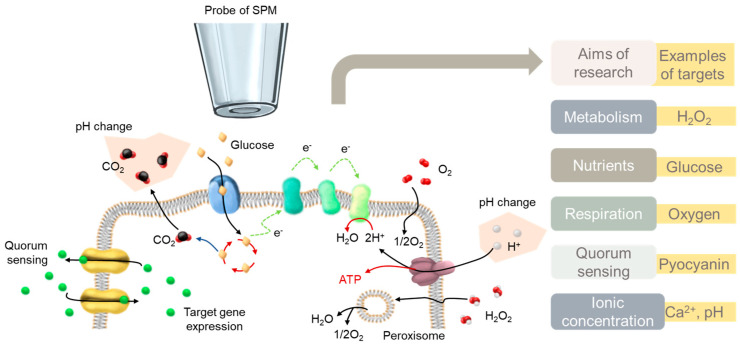
Scanning electrochemical probe−based techniques, such as SECM, are employed to explore representative indicators in bacteria and biofilm research.

Figure 1 illustrates the research objectives associated with the in situ and operando electrochemical analysis of bacteria using scanning probes. Much of the research focused on physiological characterizations, such as quorum sensing molecules (QS), electroactive metabolites, enzymes, oxygen consumption, ion concentrations, and pH values [54,61,62]. Mapping the oxygen consumption of bacteria or a single cell is an important issue for estimating bacteria activity. The detection of a pH value is also important because the pH value around the biofilm will change with biofilm metabolism. Understanding these physiological characteristics of bacteria can aid in the more efficient development of antibiotics or biofilm inhibitors.

### 3.1. SECM Probes and Complementary Techniques

**Figure 2:**
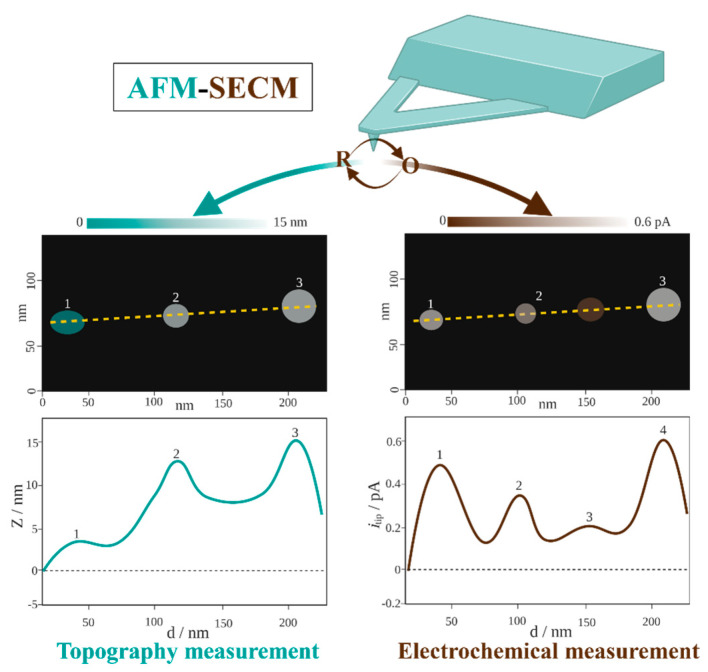
Scheme of the basic principles of topography and electrochemical measurement with AFM and SECM, respectively.

As high−resolution imaging techniques have advanced, researchers have devised combined probes that allow the simultaneous collection of multiple signals with SECM. These probes include SECM–scanning SICM [63], SECM–fluorescence microscopy [64,65], and SECM–AFM [9,43]. In particular, the use of advanced SECM–AFM (shown in Figure 2) has provided significant insights into the structures of bacteria.

Bacteria cells, which typically have a diameter of 1 µm, are suitable for AFM because of their resolution comparable to that of AFM−SECM. Unlike animal cells, bacterial cells have stiff surfaces due to their cell walls, which make AFM studies much simpler. The rigid cell wall is a crucial characteristic of bacterial cells that aids in their survival under various environmental conditions. The antigenic determinants of the cell surface are species−specific and play a vital role in the interaction between bacteria and hosts [66]. Several studies have used AFM to investigate the morphology, adhesive properties, and elasticity of bacterial cells [67,68]. Additionally, AFM can perform imaging in a liquid solution without drying the sample, enabling direct observation of molecular processes [69,70,71]. In situ imaging applications can allow for direct observation of biospecific interactions with the biopolymers of the bacterial cell, drug−induced bacteria destruction, as well as bacterial growth and division.

AFM exhibits exceptional capabilities, including high spatial and energy resolution, pico−Newton scale force sensitivity, and nanometer−scale localization accuracy. Moreover, it can detect the adhesion force of individual intact living cells. AFM can directly observe structural changes in individual biomolecules, with a resolution of about 1 nm, and can work in solutions and observe biological structures in real−world settings [72,73].

**Figure 3:**
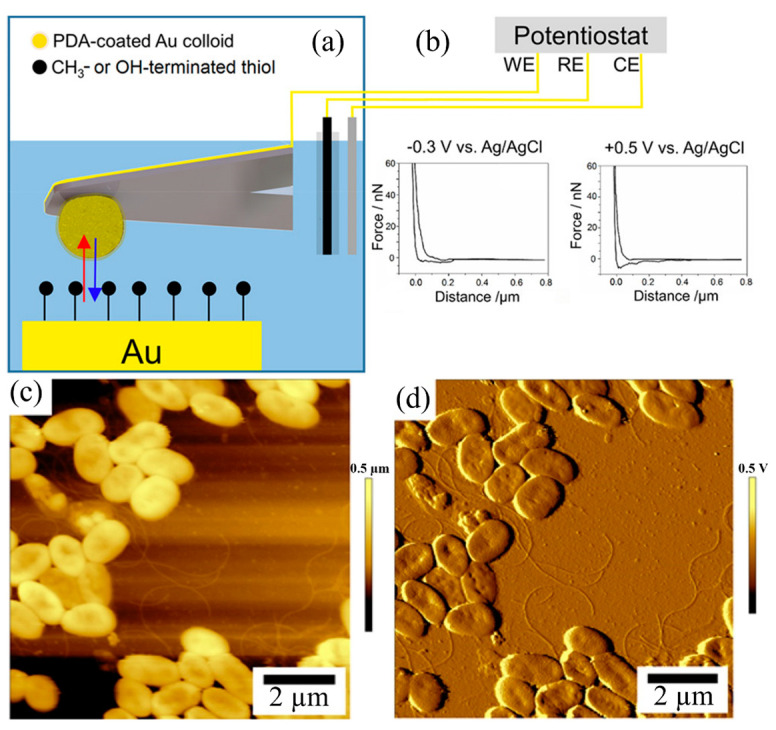
(**a**) Schematic representation of AFM−SECM probe with the function of electrochemical force spectroscopy performed on a plasma-treated gold surface in a 0.1 M KCl solution. The red and blue arrow represent the force and interaction between the probe and the sample surface. (**b**) Recorded force curves using PDA-modified colloidal probes, with biases of −0.3 V vs. Ag/AgCl and 0.5 V vs. Ag/AgCl in a 0.1 M KCl solution. (**c**) AFM topography bacterial image obtained in ambient conditions using an AFM-SECM probe. (**d**) Deflection bacterial image acquired in air using an AFM−SECM probe [43]. Copyright © 2020, American Chemical Society.

In a study by Kranz and co-workers [43], an AFM−SECM setup was utilized to explore the adhesion forces of polydopamine (PDA) applications. They investigated the bacterial adhesion force of *Pseudomonas fluorescens* on the PDA film and demonstrated the effect of surface charge on bacterial adhesion. As part of their research, they also developed a PDA-modified colloidal AFM−SECM probe with redox switchable surface properties, as shown in Figure 3. In Figure 3a, the investigation of a plasma-treated gold surface of electrochemical force spectroscopy using an AFM−SECM probe. This technique involves studying the forces between the tip of an AFM probe and the gold surface while varying the applied electrochemical potential. Figure 3b shows recorded force curves obtained using PDA-modified colloidal probes. Additionally, an AFM topography image acquired in ambient conditions is displayed in Figure 3c. This image provides a visual representation of the bacterial surface features and morphology of the sample using a silicon nitride AFM probe. Finally, a deflection bacterial image obtained in the air using a PDA-modified colloidal AFM−SECM probe is shown in Figure 3d. The deflection image allows for the characterization of the redox switchable surface properties of the sample surface. The adhesion pattern exhibits a series of adhesion peaks and ruptures, with some curves reaching up to 1.5 μm for both negatively and positively biased samples. This observation aligns with previous studies on *Pseudomonas fluorescens*, indicating possible long-range interactions attributed to the presence of flagella and fimbriae on the surface of bacterial cells.

In addition to the measurement of the adhesion force of bacteria, AFM−SECM may have significant potential in the future for the electrochemical measurement of bacteria and biofilm metabolic activity with an improved lateral resolution to topographical changes, such as adhesive coatings for cell immobilization and functional platforms for biosensors.

**Figure 4:**
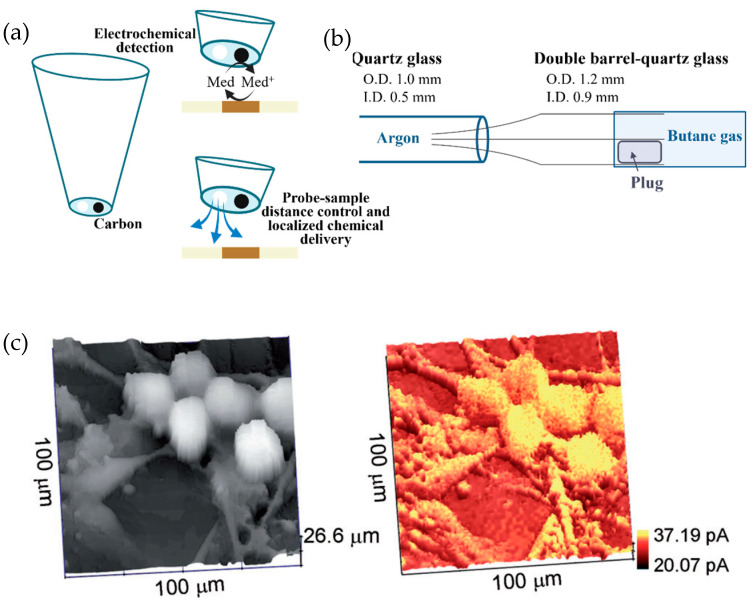
(**a**) The principle of combined SECM−SICM measurement with a double-barrel carbon nanoprobe. (**b**) Schematic illustration of the fabrication method of the double-barrel carbon nanoprobe. (**c**) Simultaneous topographic and electrochemical images of the dendritic structures of living sensory neurons [76]. Copyright © John Wiley & Sons, Inc.

SICM is a non-contact topographical analysis method that shows great potential. This technique involves injecting specific electrolytes into a glass/quartz nanopipette and then insulating and exposing the ring electrode at the capillary end [74]. SICM−SECM probes typically feature double barrels, with one barrel filled to control the SICM distance and the other containing an SECM carbon electrode to measure the uptake of the molecules of interest (see Figure 4a). Due to its precise distance control and high spatial and temporal resolutions, the combined SICM−SECM imaging method has received a lot of attention for studying dynamic biological processes. However, several challenges still exist in creating appropriate SICM−SECM probes, including achieving controlled geometry and reproducibility [75].

A simplified fabrication method for a double-barrel SICM−SECM probe with a high success rate and a rapid fabrication time (<2 min) was recently presented by Takahashi et al. [76]. Figure 4a,b illustrate the combined SECM−SICM measurement concept using double-barrel carbon nanoprobes, with effective radii of the two barrels measuring less than 50 nm and the probe’s full radius measuring less than 100 nm. SICM and SECM images showed the dendritic structures of living sensory neurons, as illustrated in Figure 4c. During this method, the SICM channel scans the target region for topographical information while the SECM electrode records the electrochemical signal of the redox mediator. In another report, the same group confirmed the capability of the probe to study the spatial distribution of neurotransmitter release and related variations in cell topography [76,77].

Unwin et al. used SICM to investigate the ionic environment of live Gram-positive and Gram-negative bacteria [63]. They employed SICM to map the ionic properties and charge environment of two live bacterial strains, namely the Gram-negative *Escherichia coli* and the Gram-positive *Bacillus subtilis*. SICM results revealed heterogeneities across the bacterial surface and significant differences between the Gram-positive and Gram-negative bacteria.

In addition, SECM can be employed in conjunction with microscopy techniques such as fluorescence microscopy. Koley et al. demonstrated the generation of a quantitative map of microbial metabolic exchange between two bacterial species, commensal *Streptococcus gordonii* and pathogenic *Streptococcus mutans*, using SECM [39]. In their study, they utilized a carbon-based potentiometric pH microsensor as an SECM chemical probe. The team also conducted fluorescence confocal imaging on Sm–Sg–Sm alginate gel biofilm with a pH molecular probe (LysoSensor yellow/blue dextran pH probe) to corroborate their SECM findings.

In the following parts, different research aims achieved by SECM and relevant technologies will be introduced in detail.

### 3.2. Measurements of O_2_ Consumption in Bacteria

Numerous studies have investigated the respiration activity of living cells to assess their metabolic vitality [78]. In these studies, probes for detecting electrochemical signals were placed near the living organism, allowing for monitoring of local oxygen concentration through oxygen reduction reactions. The microelectrode was biased, with the potential to reduce the amount of oxygen present in the buffered solution. In aerobic bacteria (i.e., microbes capable of tolerating O_2_), oxygen plays a crucial role in events such as respiration and human infection, with oxygen levels varying considerably depending on the infection site and host response [79].

**Figure 5:**
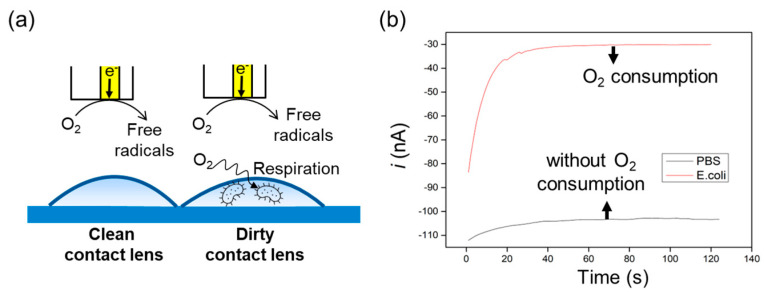
(**a**) The figure depicts a schematic diagram of a soft gold microelectrode used for measuring the currents associated with the oxygen reduction reaction. Additionally, it allows for the investigation of oxygen consumption by microbes. (**b**) Chronoamperometry was conducted to record the oxygen reduction current at a soft gold microelectrode. The measurements were taken on both clean contact lenses and contact lenses contaminated with *E. coli*. Experimental conditions: contact lenses were immersed in 0.1 x PBS solution, WE = soft gold microelectrode, QRE = Ag wire, CE = Pt wire. E = −0.8 V, delay in ADC time = 0.1 s [18]. With permission from Elsevier.

Figure 5a depicts a scheme of a Soft-Probe SECM experiment in redox competition mode, demonstrating a typical oxygen reduction process. Due to the consumption of oxygen by bacteria present in dirty contact lenses, the measured current value in chronoamperometry was lower, owing to the decrease in oxygen concentration in the PBS solution (Figure 5b) [18]. This study shows operando studies of bacterial respirations while the microbes were attached to the contact lenses. Currently, techniques for measuring living bacteria on contact lenses have been very rare and limited. Therefore, Soft-Probe SECM is a new platform for studying the microbiology of contact lens hygiene.

Furthermore, several studies have reported on the oxygen consumption by bacteria under different conditions. It is interesting to note that facultative anaerobic organisms utilize aerobic respiration, but their physiology and behavior are heavily influenced by the presence of oxygen [79]. For example, the behavior of *Pseudomonas aeruginosa*, a facultative anaerobe, has been investigated by SECM [34]. The results of the experiment indicate that the biofilm surface of *Pseudomonas aeruginosa* consumes oxygen, leading to the generation of an anoxic area. To further examine and assess the impact of oxygen consumption on biofilms, the researchers introduced an antibiotic, ciprofloxacin, into the system. Although the number of viable bacteria had decreased by a factor of 100 after the first addition of antibiotics, the hypoxic zone still existed. This is attributed to the continued bulk respiratory activity and carbon consumption despite exposure to antibiotics [80].

### 3.3. Measurements of the Interactions of Metal Ions with Bacteria

**Figure 6:**
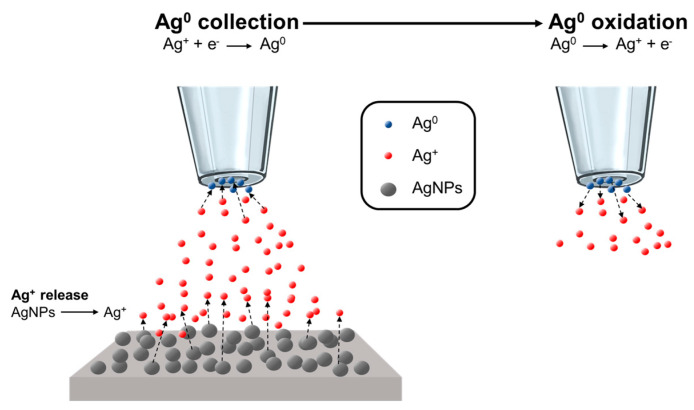
Schematic showing the release, deposition, and stripping (oxidation) of silver(I) over Ag-CFX film (represented as gray color) [44].

Exploring metal respiration by anaerobic bacteria is an important issue in various environmental processes, such as metal biogeochemical cycling, clay weathering, corrosion science, biomineralization, and production of microbial fuel cell electricity [81,82]. SECM has gained attention as a useful technique for in situ studies of the concentration profile of metal ions, including Fe, Mn, Ca, and Ag ions [42]. Additionally, SECM has been utilized to investigate suspended silver ions, silver metals, or nanoparticles embedded in Nafion or polymeric film [80]. Controlled release of silver from the film can cause the production of reactive oxygen species (ROS), resulting in cell apoptosis. Figure 6 demonstrates that SECM can effectively define the research on the kinetics of Ag release from the antibacterial film [44]. The silver is incorporated within the silver-fluoropolymer (Ag-CFX) thin films, which are designed as promising antimicrobial coatings. SECM experiments in combination with anodic stripping voltammetry (ASV) were carried out to study the release mechanism of silver(I) from the embedded silver nanoparticles (AgNPs). The antimicrobial properties of Ag-CFX are confirmed against *Pseudomonas fluorescens*, with differences observed in biofilm density and bacterial morphology for pristine and swollen films. 

### 3.4. Detection of Hydrogen Peroxide 

The detection of hydrogen peroxide can be associated with significant biological phenomena, such as catalase enzyme function, the interplay between co-cultured bacteria, metabolic activity, and the defense response against other microbial threats.

**Figure 7:**
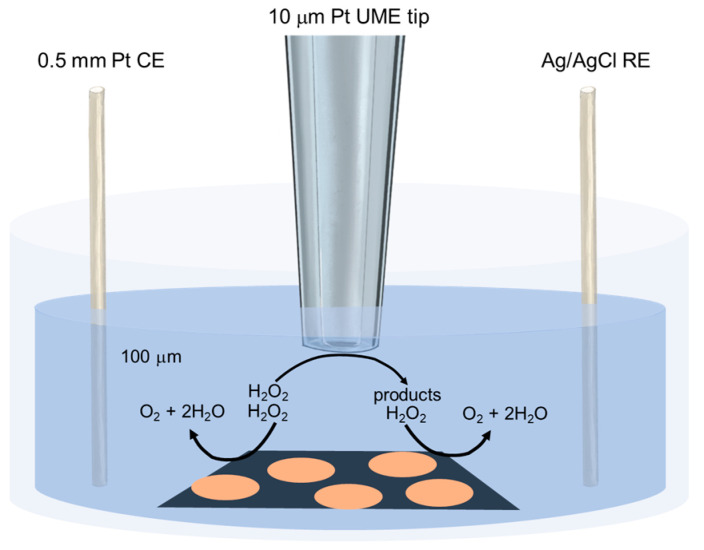
The figure illustrates a schematic representation of a real-time measurement using SECM to observe the decomposition of hydrogen peroxide by a bacterial biofilm (represented as orange color) on a substrate (represented as dark blue color) [53].

All host animals instinctively protect themselves against bacterial colonization from inappropriate or pathogenic microorganisms by activating the immune system or generating bactericides (e.g., hydrogen peroxides). Therefore, it is essential to study catalase activity to determine if bacteria can successfully colonize host tissues. Figure 7 depicts how SECM can be used to determine in real-time the catalase activity of the *Protebacteria Vibrionaceae* biofilms [53]. The decomposition of 1 mM hydrogen peroxide on the surface of Biofilms of *Protebacteria Vibrionaceae*, associated with catalase activity on the biofilm, can be easily observed in SECM without the addition of excess dyes or perturbations. 

**Figure 8:**
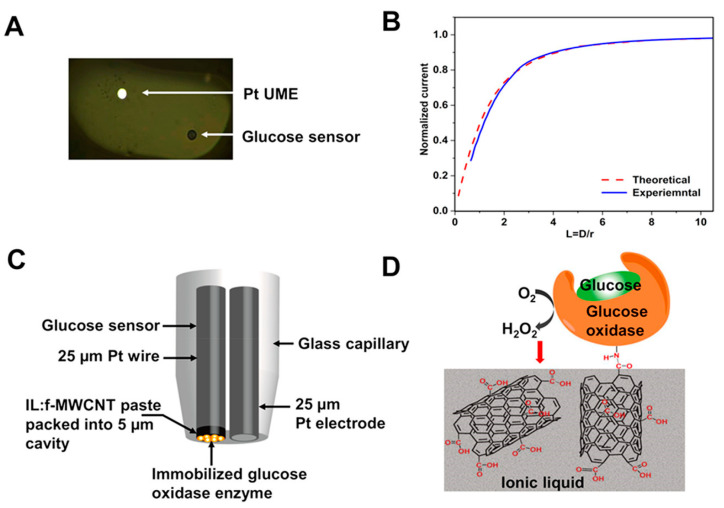
(**A**) An optical microscopic image displaying the new dual-tip SECM probe showing the Pt ultramicroelectrode (UME) and the glucose sensor. (**B**) A negative feedback approach curve was obtained with the new dual-tip SECM probe in a 1.0 mM ferrocene methanol solution fitted with the theoretical approach curve of the electrode with the same dimensions. (**C**) Schematics showcasing the configuration of the new SECM probe, including the unmodified Pt UME and the glucose UME. (**D**) A schematic representation (not drawn to scale) depicting the covalently attached glucose oxidase enzyme onto the f-MWCNT (functionalized multi-walled carbon nanotubes), which is exposed to the surface of the IL–f-MWCNT matrix packed into the etched cavity of the Pt UME [47]. Copyright © 2020, American Chemical Society.

Compared to the conventional SECM probe, the dual-scanning electrochemical microscopy probe has advantages in multi-target detection while mapping the 3D microenvironment of analytes (Figure 8A,C). In Figure 8, the dual-tip glucose-sensing SECM probe was used to measure the local glucose consumption of *Streptococcus mutans* biofilms by covalently immobilizing the glucose oxidase (GOD) enzyme (Figure 8D) [47]. A negative feedback approach curve was obtained with the new dual-tip SECM probe in ferrocene methanol solution fitted with the theoretical approach curve of the electrode with the same dimensions. This result shows the reliability of the probe (Figure 8B). The GOD was immobilized in a matrix of functionalized multi-walled carbon nanotubes (f-MWCNTs) and 1-butyl-4-methyl pyridinium hexafluorophosphate (ionic liquid) packed in the etched Pt ultramicroelectrode (Figure 8D). When GOD oxidized glucose, H_2_O_2_ was produced as one of the detectable products by the dual SECM probe, allowing the calculation of glucose concentration.

### 3.5. pH and ROS Measurement

The growth and attachment of bacteria are strongly influenced by the pH of their microenvironment [83]. To investigate the impact of local pH changes mediated by bacteria on the integrity of dental resin composite materials in the oral cavity, an SECM-based potentiometric pH microsensor was developed by Koley and co-workers [49]. The pH fluctuations of the biofilm formed by multiple strains of bacteria were monitored, revealing a decrease in pH at different stages of biofilm formation. This allowed the simulation of the dental decay process in vitro.

In another study conducted by Chaplin and co-workers, SECM was used to monitor the pH value and ROS formation near the surface of the *Pseudomonas aeruginosa* biofilm grown on a conductive surface under cathodic conditions [48]. At low applied potentials on the conductive surface, the effect of the oxidant produced on the bacterial perforation is insignificant. However, when the conductive surface was biased with 1 V, hydrogen peroxide and ROS could form on it and were detected by SECM. This enabled the authors to observe the association between bacteria viability on a conductive surface and how it was affected by H_2_O_2_ and ROS, providing a theoretical explanation for their findings [48].

### 3.6. The Research on Quorum Sensing

Bacterial biofilms can support the coexistence of numerous bacterial species, which communicate with each other via short-range chemical signals [84]. To comprehensively grasp this communication, it is crucial to explore the mechanisms underlying short-range signaling between bacteria. Quorum sensing (QS) is one of the most essential mechanisms that facilitate biofilm formation, wherein communication occurs through a population density-dependent stimulus and response system between bacterial aggregates [85]. The QS molecular formula varies depending on the bacterial type. For example, *Pseudomonas aeruginosa* produces pyocyanin (PYO), an important metabolite that not only inhibits other microorganisms but also maintains oxidative homeostasis and regulates biofilms [86]. The suppression of QS molecule secretion can hinder biofilm formation. Consequently, real-time mapping of PYO concentration using SECM in the SG/TC mode has been conducted in the previous literature [54].

### 3.7. Direct Biofilm Imaging Using a Soft-Probe SECM

The applications of conventional SECM to biological samples can be complicated by the intricate structure and morphology of such samples, resulting in difficulties with experimental procedures and data interpretation. To address these issues, Soft-Probe SECM employs a contact mode scanning approach that simplifies experimental procedures and maintains a constant working distance without requiring any additional hardware or software implementation [20,27].

**Figure 9:**
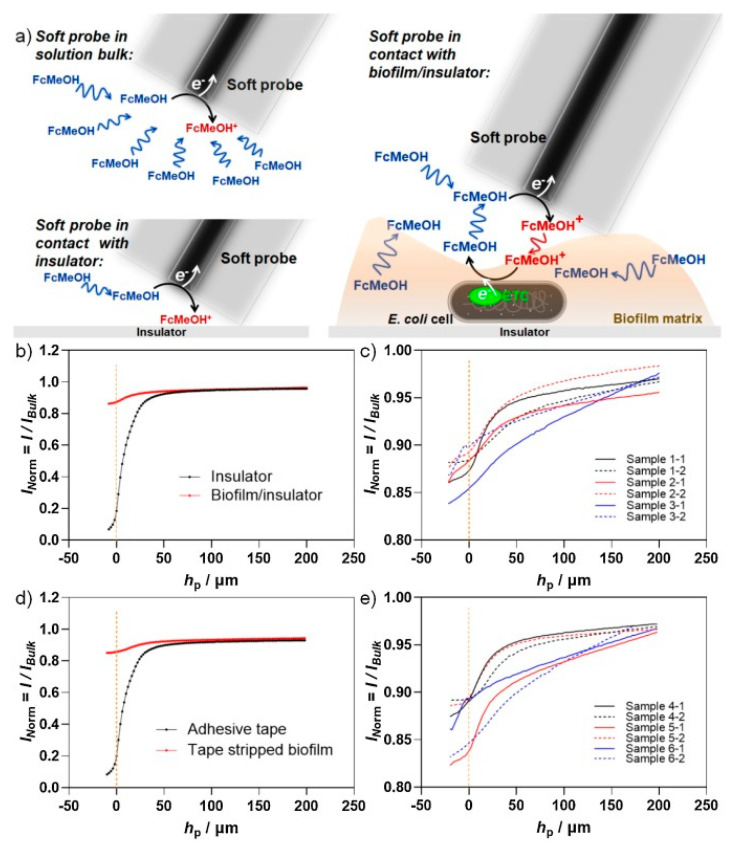
SECM feedback mode approach curves on *E. coli* biofilm. (**a**) A schematic illustration demonstrating the diffusion of FcMeOH (ferrocene methanol, represented as blue color) to the microelectrode in different scenarios: unhindered diffusion in the bulk solution when the soft probe is away from any insulator (I_Norm, bulk_), hindered diffusion when the soft probe is close or in contact with a smooth insulator such as glass (*I*_Norm,insulator_ = →0), hindered diffusion with mediator regeneration when the soft probe contacts the biofilm (*I*_Norm,insulator_ < *I*_Norm,biofilm/insulator_ < *I*_Norm,bulk_). FcMeOH was regenerated by the *E. coli* biofilm, forming FcMeOH^+^, represented as red color. (**b**) Approach curves obtained over glass and the biofilm/glass interface. (**c**) Two locally separated approach curves obtained over each of the three identical biofilms grown on glass (N_Sample_ = 3). (**d**) Approach curves over the adhesive tape and tape-stripped biofilm surface layer on adhesive tape. (**e**) Two locally separated approach curves obtained over each of the three identical tape-stripped biofilm surface layers on adhesive tapes (N_Sample_ = 3). Experimental details: *E*_T_ = 0.5 V, probe translation speed = 5 μm/s, step size = 2 μm, 2.5 mM FcMeOH in 100 mM PBS (pH = 7.4) [25]. Copyright © 2023, American Chemical Society.

Darvishi et al. reported the complementary readout of SECM images with fluorescence imaging [25]. They studied micrometric electrochemical imaging of *Escherichia coli* biofilms using Soft-Probe SECM, as shown in Figure 9. Figure 9a demonstrates the diffusion of FcMeOH to the microelectrode in three different scenarios: unhindered diffusion in the bulk solution when the soft probe is away from any insulator, hindered diffusion when the soft probe is close or in contact with a smooth insulator such as glass, and hindered diffusion with mediator regeneration when the soft probe contacts the biofilm. Since the FeMeOH could be regenerated from the biofilm, the authors also performed SECM feedback mode approach curves on *E. coli* biofilm, glass adhesive tape, and tape-stripped biofilm surface layer to observe the regenerated currents (Figure 9b-e). They found out the biofilm could be detected through SECM because of the FcMeOH reaeration currents observed. Additionally, they used SECM imaging and crystal violet staining of the surface layers of *Escherichia coli* biofilms and entire biofilms, respectively. They reported fluorescence images of whole crystal violet-stained biofilms on the glass as a complementary detection, showing biofilm biomass in different stages of biofilm maturation.

### 3.8. Characterization of the Growth of Bacteriogenic Metal Nanoparticles

The chemical method of nanoparticle production has been extensively researched, but its use of toxic reactants and organic solvents is detrimental to the environment. On the contrary, the use of microorganisms for nanoparticle synthesis is considered safer and more environmentally friendly [87]. Several microorganisms have been explored as potential cell factories for both intracellular and extracellular synthesis of various nanoparticles. SECM is an ideal tool for characterizing biosynthesized nanoparticles, as it allows for measurements in a biologically friendly environment.

**Figure 10:**
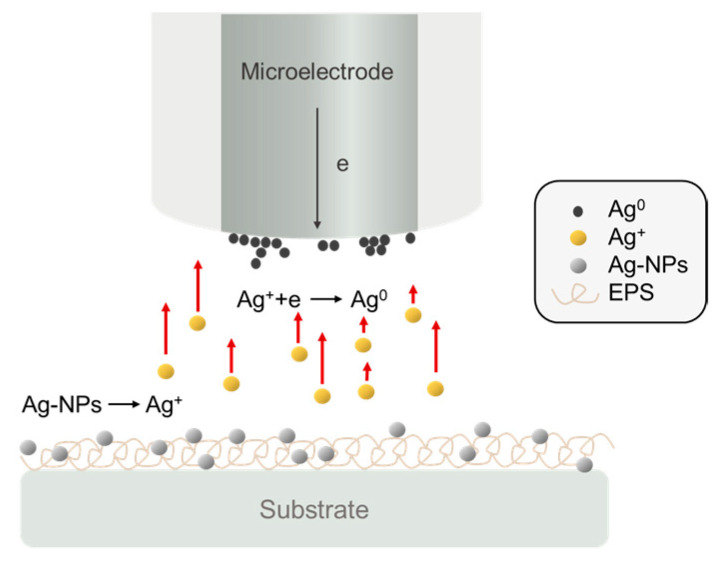
Schematic of the release of Ag(I) species from the AgNPs–EPS substrate and collection at the SECM tip [56].

In the SECM study depicted in Figure 10, the release of biosynthesized metallic nanoparticles was examined [56]. The researchers biosynthesized AgNPs using *Klebsiella oxytoca*, which produces a branched EPS during growth. Under aerobic and anaerobic conditions, AgNO_3_ was added to the bacteria culture, resulting in the formation of AgNPs embedded in EPS (AgNPs–EPS). Anodic stripping voltammetry with SECM was employed to assess the release of silver(I) species from the various AgNPs–EPS types. Consequently, SECM measurements facilitated the acquisition of information on the kinetics of silver ion release from AgNPs–EPS and their concentration profiles at the substrate/water interface.

## 4. Conclusions and Future Perspectives

In conclusion, in situ and operando electrochemical analysis performed by scanning probes in hydrophilic redox mediators or electrolytes facilitates the study of bacteria. Various types of information can be uncovered using scanning electrochemical microscopy, such as biological or electrochemical activity, respiration burst, pH levels, hydrogen concentration, electron transfer pathways, and nanoparticle biosynthesis. Several supplementary techniques have been created to address limitations in SECM and other scanning probe microscopy. These techniques aim to improve factors such as the distance between the sample and the probe, resolution, image processing, and scanning stability. These developments have expanded the capabilities of SECM and improved its performance, making it a valuable tool for studying electrochemical processes at surfaces. Specifically, the use of SECM for bacteria detection has shown great promise and has the potential to provide a powerful tool for the study of bacterial physiology and the detection of bacterial infections. With the emergence of new technologies such as artificial intelligence (AI) and the Internet of things (IoT), SECM is expected to continue to evolve and gain popularity in the future. A potential approach to improve image quality in SECM is the utilization of AI-assisted image fusion techniques, as suggested by T.-E. Lin and colleagues. This method involves combining optical microscopic images with SECM images, which holds promise for enhancing the overall quality of the resulting images [17].

## Figures and Tables

**Table 1:** The latest studies that used SECM-based tools for the analysis of bacteria and biofilms in recent years.

Research Goals	Redox Mediators or Solutions Added	Ref.
O_2_ consumption of *Pseudomonas aeruginosa*	(ferrocenylmethyl)trimethylammonium ion (FcMTMA^+^)	[34]
Fe, Mn, and O_2_ consumption of *Shewanella oneidensis*	Tris-acetate buffer, Ru(NH_3_)_6_Cl_3_	[35]
O_2_ consumption, antimicrobial mechanism of Ag^+^ in *Escherichia coli*	Electrolytes with NaNO_3_ and glucose	[36]
H_2_ consumption of *Shewanella oneidensis*	M1 solution prepared by the authors	[37]
pH and release of Ca^2+^ against *Streptococcus mutans*	Artificial saliva	[38]
Metabolic interactions of *Streptococcus gordonii* and pathogenic *Streptococcus mutans*	Artificial saliva and sugar	[39]
Calcification process of *Sporosarcina pasteurii*	Brine solution containing urea	[40]
Copper concentration near *Escherichia coli* biofilm	Hydroxymethyl ferrocene	[41]
*Metallosphaera cuprina* associated with Fe^2+^ consumption	FcMeOH or FeCl_2_	[42]
Adhesion of *Pseudomonas fluorescens*	KCl solution	[43]
Antimicrobial efficiency of silver-fluoropolymer (Ag-CFX) films against Pseudomonas fluorescens	Phosphate-buffered saline (PBS)	[44]
Hydrogen peroxide production of *Streptococcus gordonii* and interaction with Aggregatibacter actinomycetemcomitans	Chemically defined medium (CDM) culture solution with glucose	[45]
Hydrogen peroxide produced by *Streptococcus gordonii*	Glucose and artificial saliva solution	[46]
Glucose consumption of *Streptococcus mutans*	Artificial saliva solution and ferrocyanide	[47]
pH, ROS measurement, and bacteria attachment and poration of *Pseudomonas aeruginosa*	PBS, Fe(CN)_6_^3–/4–^ and Ru(NH_3_)_6_^3+/2+^	[48]
pH changes in dental-plaque-derived multi-species biofilm	Artificial saliva and FcMeOH	[49]
Characterization of a 3D-printed hydrogel with *Streptococcus mutans* and *Escherichia coli*	FcMeOH, phosphate-buffered saline (PBS)	[50]
Electron transfer train of ampicillin-resistant *Escherichia coli* on the tape	FcMeOH	[25]
Toll-like receptor array and its interaction with *Escherichia coli*	Ferrocene derivatives and electrolytes	[51]
Interaction of toll-like receptor 5 and *Salmonella typhimurium* and *Bacillus subtilis*	K_4_Fe(CN)_6_	[52]
Catalase activity of γ-Protebacteria-*Vibrionaceae* biofilms	Artificial seawater	[53]
*Pseudomonas aeruginosa* quantifies pyocyanin of QS	FcMeOH	[54]
Production of tellurium metal nanoprecipitates by *Rhodobacter capsulatus*	Phosphate-buffered saline (PBS) and lawsone	[55]
AgNPs biosynthesized by a *Klebsiella oxytoca*	K_3_IrCl_6_	[56]
Redox properties of *Shewanella oneidensis*	FcMeOH	[57]
*Corynebacterium matruchotii* fitness enhancement in adjacent *streptococci mitis*	FcMeOH	[58]
The pH of biocementation induced by *Sporosarcina pasteurii*	Brine solution and urea	[59]
Biofilm formation of *Shewanella oneidensis*	Ru(NH_3_)_6_Cl_3_	[60]
Bacteria contamination in contact lenses, including *Escherichia coli, Diphtheroid, Pseudomonas aeruginosa, Staphylococcus aureus, Fusobacterium nucleatum*, Unidentified Gram(+) *Bacilli*	Phosphate-buffered saline (PBS), FcMeOH	[18]

## Data Availability

Not applicable.
